# Fast‐acting insulin aspart in people with type 2 diabetes: Earlier onset and greater initial exposure and glucose‐lowering effect compared with insulin aspart

**DOI:** 10.1111/dom.13767

**Published:** 2019-06-10

**Authors:** Thomas R. Pieber, Eva Svehlikova, Martina Brunner, Inge B. Halberg, Karen Margrete Due Thomsen, Hanne Haahr

**Affiliations:** ^1^ Division of Endocrinology and Diabetology, Department of Internal Medicine Medical University of Graz Graz Austria; ^2^ CF Clinical Research Center, Center for Medical Research Medical University of Graz Graz Austria; ^3^ Novo Nordisk Søborg Denmark; ^4^ Novo Nordisk Aalborg Denmark

**Keywords:** clinical trial, insulin therapy, pharmacodynamics, pharmacokinetics, phase I‐II study, type 2 diabetes

## Abstract

**Aims:**

To investigate the pharmacokinetic/pharmacodynamic properties of fast‐acting insulin aspart (faster aspart) versus insulin aspart (IAsp) in people with type 2 diabetes (T2D).

**Materials and methods:**

In a randomized, double‐blind, crossover design, 61 people with T2D usually treated with insulin ± oral antidiabetic drug(s) received single‐dose faster aspart and IAsp (0.3 U/kg) on separate visits. Blood samples for pharmacokinetic assessment were collected frequently until 12 hours post‐dose. Glucose‐lowering effect was determined in a euglycaemic clamp lasting up to 12 hours post‐dose (target 5.0 mmol/L).

**Results:**

The serum IAsp pharmacokinetic profile and glucose‐lowering effect profile were shifted to the left for faster aspart versus IAsp. Least squares mean (± SE) onset of appearance was 3.3 ± 0.3 minutes for faster aspart, which was 1.2 minutes earlier than for IAsp (95% confidence interval [CI] −1.8;−0.5; *P* = .001). Onset of action for faster aspart was 8.9 minutes earlier (95% CI −12.1;−5.7; *P* < .001) than for IAsp. During the first 30 minutes after dosing, 89% larger IAsp exposure (ratio faster aspart/IAsp 1.89 [95% CI 1.56;2.28]; *P* < .001) and 147% greater glucose‐lowering effect (2.47 [95% CI 1.58;6.22]; *P* < .001) were observed for faster aspart compared with IAsp. Offset of exposure (time to 50% of maximum IAsp concentration in the late part of the pharmacokinetic profile) occurred earlier for faster aspart (difference faster aspart – IAsp −36.4 minutes [95% CI −55.3;−17.6]; *P* < .001). The treatment difference of faster aspart – IAsp in offset of glucose‐lowering effect (time to 50% of maximum glucose infusion rate in the late part of the glucose infusion rate profile) was −14.4 minutes (95% CI −34.4;5.5; *P* = .152).

**Conclusions:**

In people with T2D, faster aspart was associated with earlier onset and greater initial exposure and glucose‐lowering effect compared with IAsp, as previously shown in people with type 1 diabetes.

## 1 INTRODUCTION

Fast‐acting insulin aspart (faster aspart) is a novel insulin aspart (IAsp) formulation with two additional excipients: L‐arginine to ensure a stable formulation and niacinamide to provide increased early absorption after subcutaneous dosing^1^; thus, faster aspart is an insulin with ultra‐fast action to enable improved postprandial glycaemic control compared to that obtained with previously developed rapid‐acting insulin products.^2^, ^3^ In people with type 1 diabetes (T1D), faster aspart has an accelerated onset of appearance and an up to twofold larger initial insulin exposure and glucose‐lowering effect compared with IAsp.^4^, ^5^, ^6^, ^7^ So far, the pharmacokinetic/pharmacodynamic properties of faster aspart have not been investigated in people with type 2 diabetes (T2D).

As T2D is a progressive disease, most patients will eventually need insulin to achieve normoglycaemia. When oral antidiabetic drugs (OADs) alone or in combination with glucagon‐like peptide‐1 receptor agonists are no longer sufficient to achieve or maintain glycaemic control, current diabetes guidelines recommend the addition of basal insulin.^8^ Treatment can be further intensified by the addition of mealtime insulin in a basal‐bolus regimen to address postprandial glucose control.^8^ It has been shown that early and intensive intervention to control blood glucose lowers the risk of complications related to diabetes.^9^ The use of bolus insulin therapy is therefore a necessity in many people with T2D. Consequently, it is relevant to determine the pharmacological characteristics of a new mealtime insulin not only in people with T1D but also those with T2D.^10^

The aim of the present study, therefore, was to investigate the pharmacokinetic/pharmacodynamic characteristics of faster aspart versus IAsp for the first time in people with T2D.

## 2 MATERIALS AND METHODS

### 2.1 Trial design

This single‐centre (Department of Internal Medicine, Division of Endocrinology and Diabetology, Medical University of Graz, Austria), randomized, double‐blind, two‐period, crossover trial in people with T2D was performed according to the Declaration of Helsinki and Good Clinical Practice. Following local regulations, health authorities and the Independent Ethics Committee of the Medical University of Graz reviewed and approved the trial protocol. All participants provided written informed consent before any trial‐related activities were initiated. The trial was registered at ClinicalTrials.gov (NCT02933853).

### 2.2 Participants

Eligible participants were men and women aged 18 to 75 years, diagnosed with T2D for ≥12 months, with stable body mass index (BMI) <35 kg/m^2^, glycated haemoglobin (HbA1c) ≤9.5% (≤80 mmol/mol) and fasting C‐peptide ≤0.9 nmol/L (extended from <0.6 nmol/L during the trial). Participants also had to have been treated with multiple daily insulin injection therapy or continuous subcutaneous insulin infusion for ≥12 months (total insulin dose 0.3‐1.5 (I)U/kg/d and total bolus insulin dose <1.2 (I)U/kg/d) with or without metformin, insulin secretagogues, dipeptidyl peptidase‐4 (DPP‐4) inhibitors or sodium‐glucose co‐transporter‐2 (SGLT2) inhibitors.

### 2.3 Procedures

Participants attended a screening visit, an OAD washout visit (14‐21 days prior to the first dosing visit), two dosing visits (separated by 7‐42 days) and a follow‐up visit (7‐21 days after the last dosing visit). The OAD washout visit was only performed in participants being treated with insulin secretagogues, DPP‐4 inhibitors or SGLT2 inhibitors in combination with insulin with or without metformin. At the OAD washout visit, participants were asked to continue any metformin therapy at an unchanged dose throughout the trial and to terminate treatment with any other OAD(s).

At the two dosing visits, participants received single dosing of 0.3 U/kg faster aspart (100 U/mL; Novo Nordisk, Bagsværd, Denmark) or IAsp (NovoRapid® 100 U/mL; Novo Nordisk) in a randomized sequence. Both trial products were provided in blinded PDS290 pen‐injector prefilled pens (Novo Nordisk) and administered subcutaneously in a lifted skin fold of the lower abdominal wall above the inguinal area. Use of current insulin was terminated in due time to allow washout before faster aspart and IAsp administration.

Participants arrived at the clinical site at 4:30 pm the day before dosing, were served a standardized meal and started fasting from 7:00 pm. In order to assess the pharmacodynamics of faster aspart and IAsp, a euglycaemic glucose clamp was conducted with an overnight run‐in period starting at 10:00 pm. Participants received a variable intravenous infusion of human insulin [40 IU Actrapid® 100 IU/mL (Novo Nordisk) in 99.6 mL saline] or 20% glucose to obtain the plasma glucose (PG) target concentration of 5.0 mmol/L. The trial product was administered between 8:00 am and 10:00 am the next morning after PG had stabilized for ≥1 hour with minimum insulin infusion and no glucose infusion. At the time of dosing, any infusion of intravenous insulin was terminated. After PG had declined by 0.3 mmol/L (defined as onset of action), a variable intravenous glucose infusion was initiated to keep PG at the target throughout the clamp. The clamp continued for up to 12 hours after dosing, but was terminated if PG was consistently ≥11.1 mmol/L, with no requirement for intravenous glucose infusion during the previous 30 minutes. The individual clamps were performed with similar high quality across both treatments (Figure S1 and Table S1 in Appendix S1).^11^

Blood sampling for pharmacokinetics was performed frequently from 2 minutes prior to dosing until 12 hours post‐dose (Table S2 in Appendix S1).

### 2.4 Assessments

A validated IAsp‐specific enzyme‐linked immunosorbent assay with a lower limit of quantification (LLOQ) of 10 pmol/L was used to measure free serum IAsp concentrations following polyethylene glycol precipitation.

Measurement of PG concentrations during the glucose clamp was performed with a SuperGL 2 glucose analyser (Dr Müller Gerätebau GmbH, Freital, Germany) using an electrochemical method.

Serum free fatty acid (FFA) concentrations were measured using a Wako NEFA assay (Wako Diagnostics, Mountain View, California), performed on a Roche Cobas C501 analyser (Roche Diagnostics GmbH, Mannheim, Germany).

Safety assessments included adverse events (AEs), hypoglycaemic episodes, injection site reactions, laboratory safety measures, physical examination, vital signs and ECG. AEs were defined as treatment‐emergent if they had onset within the period from time of trial product administration until 7 days later. Hypoglycaemic episodes were classified according to the American Diabetes Association^12^ and in addition defined as “confirmed” if documented by PG <3.1 mmol/L with or without symptoms consistent with hypoglycaemia. Hypoglycaemic episodes were defined as treatment‐emergent if they had onset between the time of trial product administration and the subsequent administration of any insulin; however, these should occur no longer than 16 hours after trial product administration.

### 2.5 Endpoints

All pharmacokinetic and glucose infusion rate (GIR) endpoints to assess onset of exposure and glucose‐lowering effect, initial IAsp exposure and glucose‐lowering effect, offset of exposure and glucose‐lowering effect, and overall exposure and glucose‐lowering effect were defined and derived as previously described.^5^ In a blinded review of pharmacokinetic profiles, it was observed that selected profiles had pre‐dose values above the LLOQ and stayed above the LLOQ for the entire sampling period. It was therefore decided to baseline‐correct these profiles using a baseline value derived as the mean of all pre‐dose samples. After baseline correction, all pre‐dose values and negative values were set to zero. All pharmacokinetic endpoints for the baseline‐corrected profiles were derived in the same way as those for the non‐baseline‐corrected profiles.

The suppression of serum FFA concentration initially after dosing was determined by calculating the area over the baseline‐corrected serum FFA concentration‐time curve during the first hour (ΔAOC_FFA,0‐1 h_) and the first 2 hours (ΔAOC_FFA,0‐2 h_) as well as the time to reach 50% of the maximum FFA decline in the initial part of the FFA profile (t_50% max FFA decline_). Furthermore, the minimum FFA concentration (FFA_min_) was derived.

### 2.6 Statistical analysis

The sample size calculation was based on a comparison of the primary endpoint (area under the curve for serum IAsp during the first 30 minutes after dosing; AUC_IAsp,0‐30 min_) between faster aspart and IAsp in particpants with T2D, using information for an earlier faster aspart formulation from a previous trial that included both participants with T1D and T2D.^13^ A comparison of the secondary endpoint, area under the GIR curve during the first hour after dosing (AUC_GIR,0‐1 h_), between faster aspart and IAsp, using information from participants with T1D^4^ adjusted to the present population of participants with T2D, was also taken into consideration. A total of 56 completers were required to obtain at least 90% power for the detection of a treatment ratio of 1.25 with a within‐participant standard deviation of 0.35 on log‐scale for AUC_GIR,0‐1 h_. A total of 56 completers resulted in a statistical power of >99% for AUC_IAsp,0‐30 min_ assuming a ratio of 2 between faster aspart and IAsp with a within‐participant standard deviation of 0.3 on log‐scale. To account for participant withdrawals, 60 participants were planned to be randomized. Withdrawn participants or non‐evaluable participants could, however, be replaced by additional participants in order to ensure a sufficient number of completed and evaluable participants.

Statistical analyses were performed using SAS version 9.4 (SAS Institute, Cary, North Carolina). All endpoints were compared between faster aspart and IAsp using a linear mixed model, with treatment and period as fixed effects and participant as a random effect. The *P* value for the two‐sided test of no treatment difference using a significance level of 5% was estimated from the model. For FFA endpoints, baseline FFA was included as a covariate. Before analysis, AUC_IAsp_ endpoints, maximum IAsp concentration (C_max_), AUC_GIR,0‐1.5 h_, AUC_GIR,0‐2 h_ and maximum GIR (GIR_max_) were log‐transformed. Least squares means for each treatment, treatment ratios and 95% confidence intervals (CIs) were calculated on the original scale. All other endpoints were analysed on a linear scale: least squares means for each treatment, treatment differences and 95% CIs were estimated. Treatment ratios and 95% CIs were determined according to Fieller's method.^14^

## 3 RESULTS

### 3.1 Participant disposition and baseline demographics

A total of 176 individuals were screened and 61 were randomized and exposed to trial product (including 60 participants planned to be randomized plus one replacement participant). In total, 57 participants completed the trial. The safety analysis set comprised all 61 participants exposed to trial product. The full analysis set used for pharmacokinetic/pharmacodynamic analysis included 59 participants. One participant not fulfilling all inclusion criteria and therefore included in error, and one participant receiving faster aspart at both dosing visits were excluded from the full analysis set. The latter participant was replaced. Participant disposition is shown in Figure S2 in Appendix S1.

The 61 exposed participants had a mean ± SD age of 61.8 ± 7.6 years, 26.2% were women and all participants were white. The mean body weight was 87.8 ± 13.9 kg, mean BMI was 29.6 ± 3.2 kg/m^2^, mean duration of diabetes was 20.8 ± 7.8 years, and mean HbA1c was 60 ± 11 mmol/mol (7.6 ± 1.0%). At entry into the trial, 26 participants were only treated with insulin, 19 were treated with insulin + metformin, 13 were treated with insulin + metformin+ other OADs (DPP‐4 inhibitors and/or SGLT2 inhibitors) and three were treated with insulin + other OADs (DPP‐4 inhibitors and/or SGLT2 inhibitors).

### 3.2 Onset, initial exposure and initial glucose‐lowering effect

**Figure 1:**
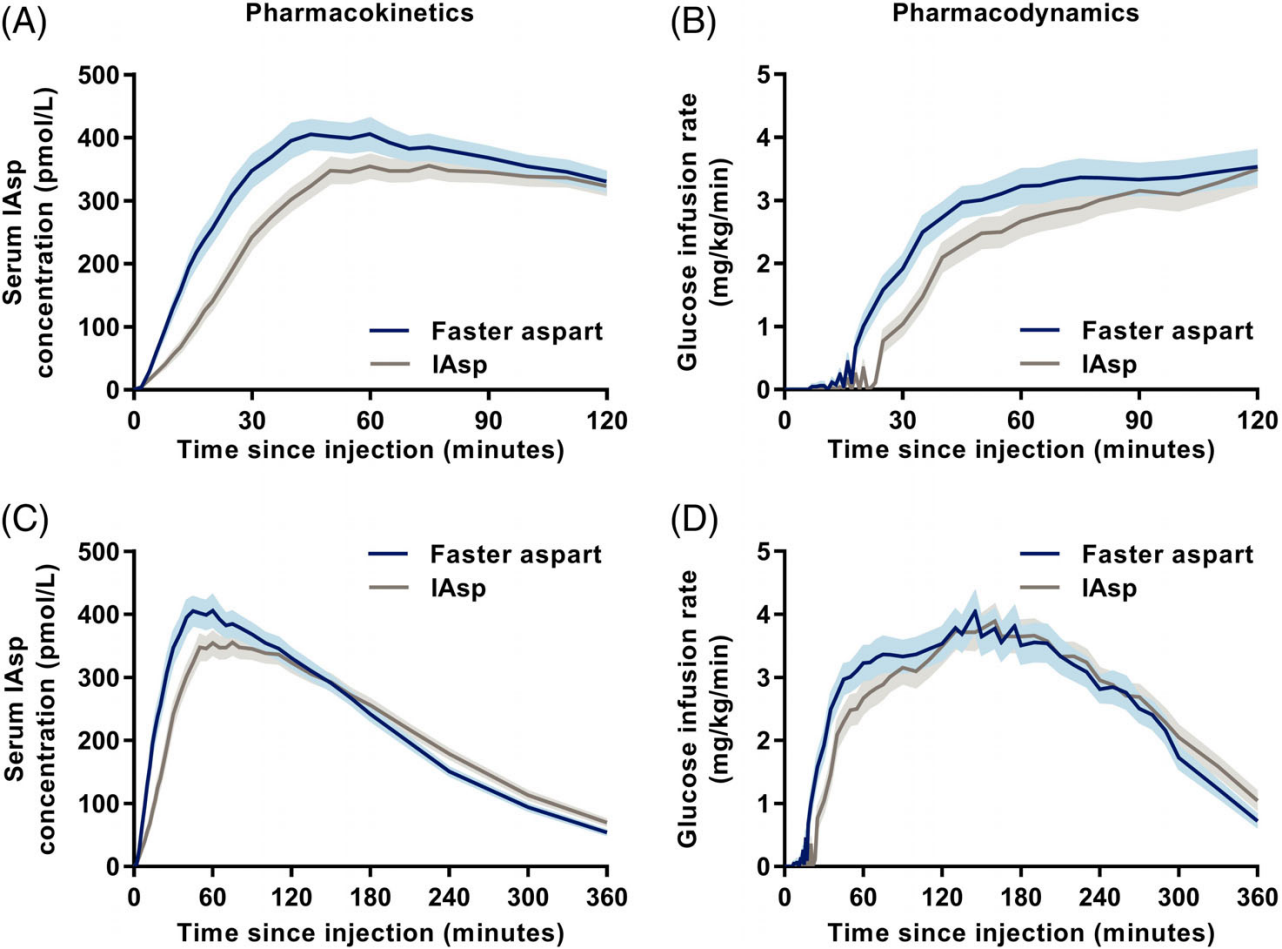
Pharmacokinetic and pharmacodynamic profiles after subcutaneous dosing of 0.3 U/kg fast‐acting insulin aspart (faster aspart) or insulin aspart (IAsp) in people with type 2 diabetes. A, Mean serum IAsp concentration‐time profiles for 2 hours and C, 6 hours after dosing, and B, mean glucose infusion rate profiles for 2 hours and D, 6 hours after dosing. Variability bands show the SEM. Number of participants: 56 for faster aspart and 59 for IAsp

The serum IAsp pharmacokinetic profile and glucose‐lowering effect profile were both left‐shifted for faster aspart compared with IAsp (Figure 1), indicating earlier onset and greater initial exposure and glucose‐lowering effect versus IAsp. Onset of appearance took place 3.3 minutes after faster aspart administration, which was 1.2 minutes earlier than for IAsp (Table 1). Furthermore, time to 50% of maximum IAsp concentration in the initial part of the pharmacokinetic profile (t_Early 50% Cmax_) happened 8.5 minutes earlier and time to maximum IAsp concentration (t_max_) happened 16.9 minutes earlier for faster aspart versus IAsp (Table 1). Likewise, onset of action and time to 50% of maximum GIR in the initial part of the GIR profile (t_Early 50% GIRmax_) took place 8.9 and 11.8 minutes earlier for faster aspart than for IAsp. Time to maximum GIR (t_GIRmax_) did not differ significantly between faster aspart and IAsp (Table 1).

**Table 1:** Onset of exposure and onset of glucose‐lowering effect for fast‐acting insulin aspart versus insulin aspart in people with type 2 diabetes

	Faster aspart^a^	IAsp^a^	Treatment ratio^b^ (95% CI)	Treatment difference^c^ (95% CI)	*P* d
**Onset of exposure**					
Onset of appearance, min	3.3 ± 0.3	4.4 ± 0.3	0.74 (0.61;0.88)	−1.2 (−1.8;−0.5)	.001
t_Early 50% Cmax_, min	19.3 ± 1.0	27.9 ± 1.0	0.69 (0.63;0.76)	−8.5 (−10.8;−6.3)	<.001
t_max_, min	70.0 ± 5.4	86.9 ± 5.2	0.81 (0.69;0.93)	−16.9 (−27.9;−5.9)	.003
**Onset of glucose‐lowering effect**					
Onset of action, min	22.4 ± 1.5	31.3 ± 1.4	0.71 (0.63;0.81)	−8.9 (−12.1;−5.7)	<.001
t_Early 50% GIRmax_, min	39.3 ± 2.4	51.1 ± 2.3	0.77 (0.66;0.89)	−11.8 (−18.1;−5.4)	<.001
t_GIRmax_, min	150.9 ± 9.0	155.6 ± 8.7	0.97 (0.84;1.12)	−4.7 (−27.5;18.1)	.680

Abbreviations: CI, confidence interval; faster aspart, fast‐acting insulin aspart; IAsp, insulin aspart; t_Early 50% Cmax_, time to 50% of maximum IAsp concentration in the initial part of the pharmacokinetic profile; t_Early 50% GIRmax_, time to 50% of maximum glucose infusion rate in the initial part of the glucose infusion rate profile; t_GIRmax_, time to maximum glucose infusion rate; t_max_, time to maximum IAsp concentration.

Number of participants: 56 for faster aspart (55 for t_max_) and 59 for IAsp.

a Data are least squares means ± standard errors.

b Faster aspart/IAsp (calculated using Fieller's method).

c Faster aspart – IAsp.

d For treatment comparison of faster aspart vs IAsp (estimated from the linear mixed model).

**Figure 2:**
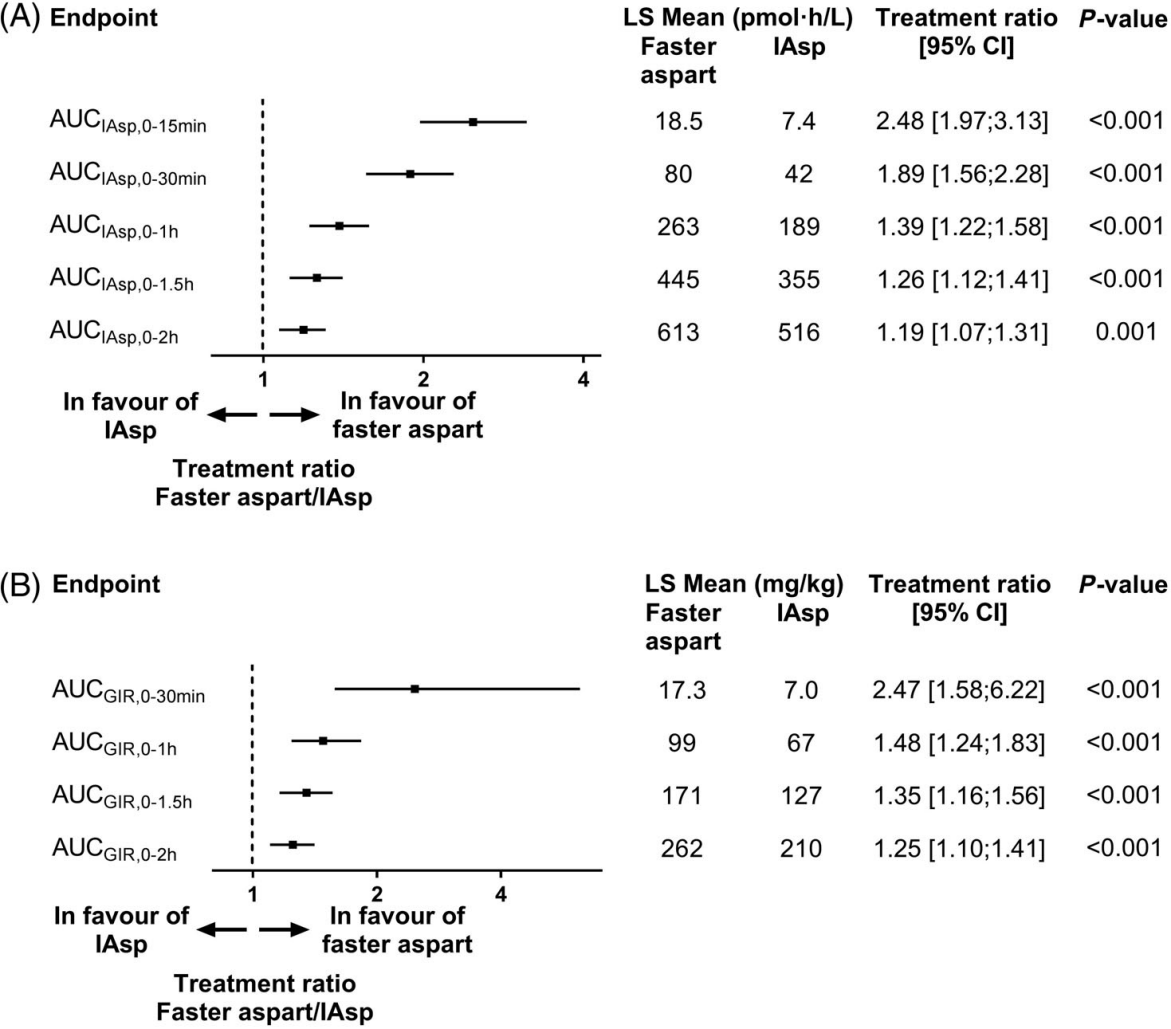
A, Initial exposure and B, initial glucose‐lowering effect for fast‐acting insulin aspart (faster aspart) versus insulin aspart (IAsp) in people with type 2 diabetes. Number of participants: 56 for faster aspart and 59 for IAsp. AUC, area under the curve; CI, confidence interval; GIR, glucose infusion rate; LS, least squares. *P* values are for treatment comparison of faster aspart vs IAsp (estimated from the linear mixed model); treatment ratio is faster aspart/IAsp (calculated using Fieller's method for AUC_GIR,0‐30 min_ and AUC_GIR,0‐1 h_)

Initial IAsp exposure and initial glucose‐lowering effect up to 2 hours were both larger for faster aspart versus IAsp (Figure 2). Within the first 30 minutes after dosing, 89% higher IAsp exposure (AUC_IAsp,0‐30 min_) and 147% larger glucose‐lowering effect (AUC_GIR,0‐30 min_) were observed for faster aspart than for IAsp.

### 3.3 Offset of exposure and glucose‐lowering effect

Offset of IAsp exposure happened earlier for faster aspart than for IAsp. This is seen from the shorter time to 50% of maximum IAsp concentration in the late part of the pharmacokinetic profile (t_Late 50% Cmax_) for faster aspart versus IAsp (treatment difference faster aspart – IAsp: −36.4 min [95% CI −55.3;−17.6], *P* < .001) and the smaller partial area under the serum IAsp pharmacokinetic profile from 2 hours (AUC_IAsp,2‐t_) for faster aspart versus IAsp (treatment ratio faster aspart/IAsp 0.88 [95% CI 0.81;0.95]; *P* = .002 [Table S3 in Appendix S1]). For time to 50% of maximum GIR in the late part of the GIR profile (t_Late 50% GIRmax_), the treatment difference of faster aspart – IAsp was −14.4 minutes (95% CI −34.4;5.5; *P* = .152). For the partial area under the GIR profile from 2 hours (AUC_GIR,2‐t_), the treatment ratio of faster aspart/IAsp was 0.91 (95% CI 0.82;1.01; *P* = .083 [Table S3 in Appendix S1]). Thus, based on the point estimates, offset of glucose‐lowering effect also took place earlier for faster aspart versus IAsp, although the treatment differences did not reach statistical significance.

### 3.4 Overall exposure and glucose‐lowering effect

The total IAsp exposure (AUC_IAsp,0‐t_) was similar for faster aspart and IAsp (treatment ratio faster aspart/IAsp 0.99 [95% CI 0.94;1.04]; *P* = .646), while C_max_ was modestly higher for faster aspart versus IAsp (1.13 [95% CI 1.02;1.24]; *P* = .018 [Table S4 in Appendix S1]). The total glucose‐lowering effect (AUC_GIR,0‐t_) and GIR_max_ were both similar for faster aspart and IAsp (1.00 [95% CI 0.92;1.08], *P* = .960 and 1.03 [95% CI 0.96;1.11], *P* = .373, respectively; [Table S4 in Appendix S1]).

### 3.5 Free fatty acids

**Figure 3:**
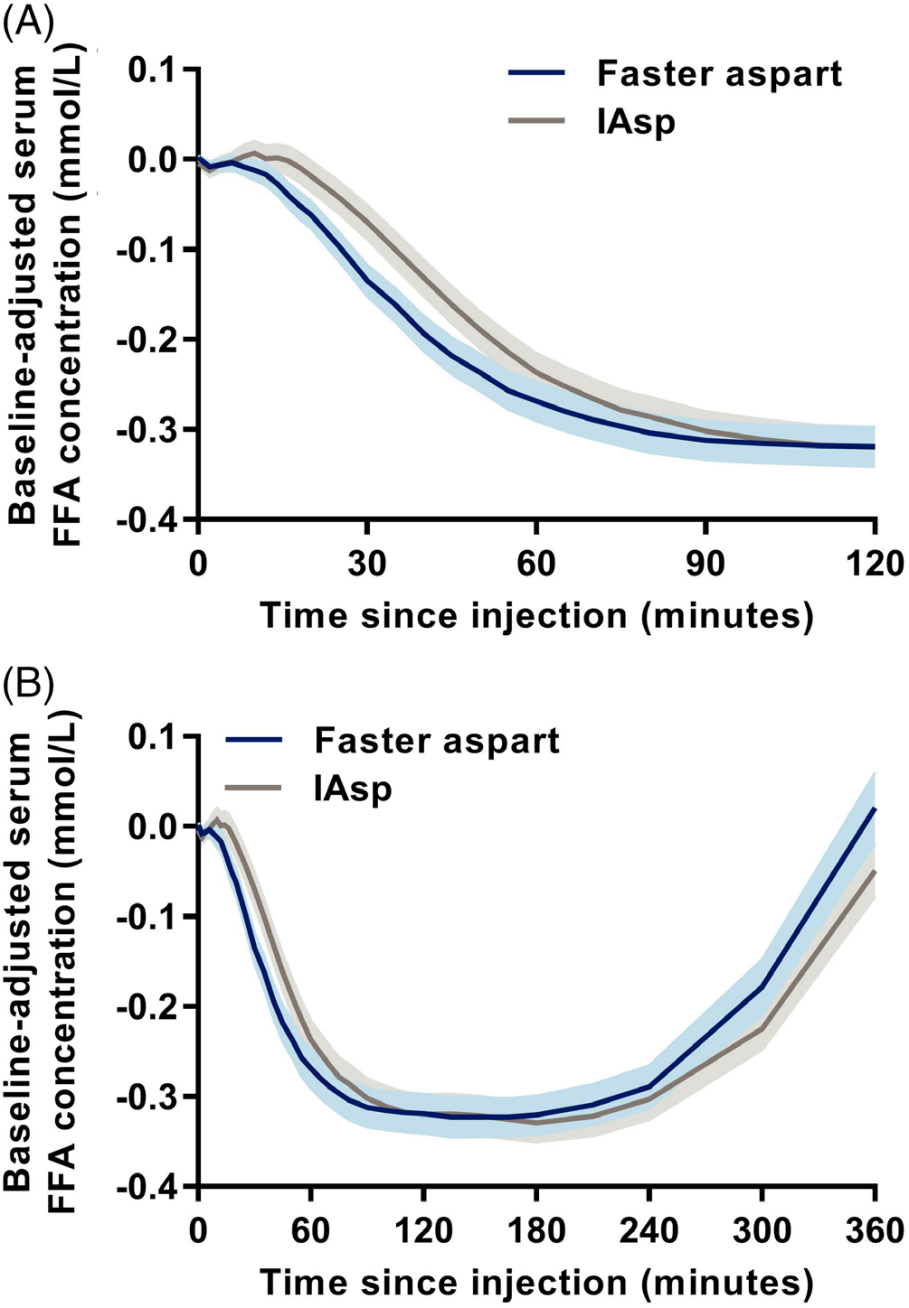
Mean baseline‐corrected serum free fatty acid (FFA) concentration for A, 2 hours and B, 6 hours after subcutaneous dosing of 0.3 U/kg fast‐acting insulin aspart (faster aspart) or insulin aspart (IAsp) in people with type 2 diabetes. Variability bands show the SEM. Number of participants: 56 for faster aspart and 58 for IAsp (one FFA profile for IAsp was excluded because of implausibly interchanging high and low values)

Faster aspart appeared to suppress FFA more quickly than IAsp, as the mean baseline‐corrected serum FFA concentration‐time profile was left‐shifted for faster aspart versus IAsp (Figure 3). ΔAOC_FFA,0‐1 h_ was 47% greater and ΔAOC_FFA,0‐2 h_ was 15% greater for faster aspart versus IAsp, while t_50% max FFA decline_ was 10.3 minutes shorter with faster aspart versus IAsp (Table 2). The mean minimum FFA concentration was 0.08 ± 0.05 mmol/L for both faster aspart and IAsp.

**Table 2:** Decrease in free fatty acids for fast‐acting insulin aspart versus insulin aspart in people with type 2 diabetes

	Faster aspart^a^	IAsp^a^	Treatment ratio^b^ (95% CI)	Treatment difference^c^ (95% CI)	*P* d
ΔAOC_FFA,0‐1 h_, mmol·h/L	0.13 ± 0.01	0.09 ± 0.01	1.47 (1.19;1.88)		<.001
ΔAOC_FFA,0‐2 h_, mmol·h/L	0.44 ± 0.02	0.38 ± 0.02	1.15 (1.06;1.24)		<.001
t_50% max FFA decline_, min	39.7 ± 2.3	50.0 ± 2.3	0.79 (0.71;0.88)	−10.3 (−14.8;−5.8)	<.001

Abbreviations: ΔAOC_FFA,0‐x_, area over the baseline‐corrected curve for free fatty acids from time zero to x; CI, confidence interval; faster aspart, fast‐acting insulin aspart; FFA, free fatty acids; IAsp, insulin aspart; t_50% max FFA decline_, time to reach 50% of the maximum decline in free fatty acids.

Number of participants: 56 for faster aspart and 58 for IAsp (one FFA profile for IAsp was excluded as a result of implausibly interchanging high and low values).

a Data are least squares means ± SE.

b Faster aspart/IAsp (calculated using Fieller's method).

c Faster aspart – IAsp.

d For treatment comparison of faster aspart versus IAsp (estimated from the linear mixed model).

### 3.6 Safety

Faster aspart and IAsp were well tolerated, and no safety issues were identified in the present trial. A total of 27 treatment‐emergent AEs (16 with faster aspart and 11 with IAsp) were reported. The majority of AEs were mild in intensity and assessed as unlikely to be related to treatment. There were four serious AEs in one participant (fall, skull fracture, subdural haematoma and cerebral haematoma), all reported 5 days after treatment with faster aspart and assessed by the investigator to be unlikely to be related to treatment. The participant recovered with sequelae. No clinically relevant findings were made in safety laboratory assessments, vital signs, physical examination or ECG, and no injection site reactions or treatment‐emergent hypoglycaemic episodes were reported during the trial.

## 4 DISCUSSION

The present study is the first to compare the pharmacokinetic/pharmacodynamic characteristics between faster aspart and IAsp in people with T2D. The main findings were that the serum IAsp pharmacokinetic profile, the glucose‐lowering effect profile and the FFA profile were all left‐shifted for faster aspart versus IAsp. Faster aspart provided earlier onset of appearance, earlier onset of action and approximately twofold greater exposure and 2.5‐fold greater glucose‐lowering effect within the first 30 minutes after dosing compared with IAsp. Furthermore, offset of exposure happened earlier for faster aspart versus IAsp, while the numerical treatment difference in offset of glucose‐lowering effect favouring faster aspart was not statistically significant. Finally, suppression of FFA concentration in response to insulin administration occurred earlier with faster aspart than with IAsp.

The overall left‐shift of the pharmacokinetic/pharmacodynamic profiles with faster aspart versus IAsp in the present study in participants with T2D was comparable to that seen in people with T1D.^5^, ^6^, ^15^ It has been reported that the absorption kinetics of human insulin are slower in people with T2D than in those with T1D^10^, ^16^; therefore, it is interesting that absolute values for onset of appearance, t_Early 50% Cmax_, onset of action and t_Early 50% GIRmax_ for faster aspart in the present study in people with T2D are similar to those seen in people with T1D.^5^, ^6^, ^15^ This indicates that the pharmacological characteristics of faster aspart immediately after dose administration are independent of type of diabetes.

The further left‐shift of the pharmacokinetic/pharmacodynamic profiles seen with faster aspart represents another step towards reproducing the physiological insulin secretion after a meal. In a 26‐week phase III trial, the more physiological insulin profile of faster aspart was shown to translate into improved early postprandial glycaemic control within the first hour after meal ingestion compared with IAsp when used in a basal‐bolus regimen in people with T2D inadequately controlled on OADs and basal insulin.^17^ It was recently shown that the improved postprandial glucose control seen with faster aspart during the first hour after a meal is attributable to a combination of greater early suppression of endogenous glucose production and greater increase in glucose disappearance^7^; thus, the mechanisms explaining the reduced postprandial glucose concentration with faster aspart mimic the physiological dual response to increased circulating insulin after meal initiation, consisting of a rapid reduction in hepatic glucose output combined with an increased peripheral glucose uptake.^2^ The present finding of earlier suppression of FFA concentration with faster aspart versus IAsp is intriguing because reduced FFA availability to the liver may lead to suppression of hepatic glucose output via decreased hepatic FFA oxidation and, in turn, inhibition of gluconeogenesis.^18^, ^19^, ^20^ It was recently suggested, based on a study in people with T1D, that the greater early suppression of endogenous glucose production with faster aspart might be partly attributable to larger initial suppression of the FFA level in the circulation.^7^ It follows that the earlier suppression of FFA in the present study in T2D may contribute to the early improved postprandial glycaemic control seen with faster aspart versus IAsp.^17^

For t_max_, t_GIRmax_ and offset of exposure and glucose‐lowering effect, the present results indicate generally longer tails of the faster aspart and IAsp pharmacokinetic/pharmacodynamic profiles in T2D than in T1D, as observed previously.^5^ It is, however, important to note that the left‐shift of the late part of the pharmacokinetic/pharmacodynamic profiles seen with faster aspart versus IAsp in the present study was at least as large as that seen in people with T1D.^5^ While t_Late 50% Cmax_ occurred 36.4 minutes earlier for faster aspart than for IAsp in T2D in the present study versus 12.2 minutes earlier in T1D in the previous study, the corresponding values for t_Late 50% GIRmax_ were 14.4 versus 14.3 minutes.^5^ The 14.3 minutes‐earlier offset of glucose‐lowering effect for faster aspart than for IAsp in a pooled analysis of 218 people with T1D was highly significant (*P* < .001).^5^ In contrast, the 14.4‐minutes earlier offset of glucose‐lowering effect for faster aspart than for IAsp in the present study was not statistically significant. This may be attributable to the lower number of participants in the present study combined with a generally higher variability in glucose‐lowering effect in T2D compared with T1D.

Earlier offset of exposure and glucose‐lowering effect with faster aspart versus IAsp might improve the balance between the glucose load from a meal and the exogenous insulin absorption during the late postprandial phase, leading to lower risk of late postprandial hypoglycaemia. Accordingly, in a report of two phase III trials investigating faster aspart versus IAsp used in a basal‐bolus treatment regimen in people with T1D, a significantly reduced hypoglycaemia rate was shown with faster aspart versus IAsp from 3 to 4 hours after a meal in one trial.^21^ In contrast, there is also a risk that the offset could occur too fast, which would lead to insufficient levels of circulating insulin during the late postprandial phase.^2^ However, this issue may be less relevant in people with T2D because the latter part of the insulin pharmacokinetic profile appears to be generally shifted to the right compared to that in people with T1D.^10^, ^16^

A limitation to the accurate understanding of t_Early 50% Cmax_ and t_Late 50% Cmax_ was that C_max_ was significantly higher for faster aspart than for IAsp.^22^ Importantly, however, the higher C_max_ for faster aspart would presumably imply an artificial increase in t_Early 50% Cmax_ and t_Late 50% Cmax_. Thus, if C_max_ had been at the same level for faster aspart and IAsp, t_Early 50% Cmax_ and t_Late 50% Cmax_ might have been even shorter for faster aspart compared to IAsp.

A potential limitation to the generalizability of the present study was the inclusion of people with relatively progressed T2D. Since bolus insulin should be introduced as soon as basal insulin alone is insufficient to maintain glycaemic control, faster aspart is more relevant for patients with shorter duration of diabetes than those included in the present study.^8^ Along these lines, it is interesting that a phase III trial has shown that faster aspart, applied with a simple titration algorithm, can be added to a basal‐only regimen in people with T2D inadequately controlled on basal insulin plus OAD(s), with satisfactory outcome.^23^

In conclusion, the present study shows that in people with T2D, faster aspart provides earlier onset and greater initial exposure and glucose‐lowering effect compared with IAsp. The present findings in people with T2D are in accordance with the results of previous studies comparing the pharmacokinetic/pharmacodynamic characteristics of faster aspart and IAsp in people with T1D. The present study therefore supports the potential of faster aspart to improve postprandial glucose control compared with IAsp also in people with T2D.

## CONFLICT OF INTEREST

T.R.P. has received research support from AstraZeneca and Novo Nordisk, has served on advisory panels for AstraZeneca, Bristol‐Myers Squibb, Eli Lilly, Novo Nordisk and Roche Diabetes Care, and is an employee of CBmed‐Center for Biomarker Research in Medicine (a public owned research company). I.B.H., K.M.D.T. and H.H. are employees and shareholders of Novo Nordisk. E.S. and M.B. declare no conflicts of interest.

## AUTHOR CONTRIBUTIONS

T.R.P. and I.B.H. contributed to the trial design, conduct/data collection, analysis and writing the manuscript. E.S. and M.B. contributed to conduct/data collection, analysis and writing the manuscript. K.M.D.T. contributed to analysis and writing of the manuscript. H.H. contributed to the trial design, analysis and writing of the manuscript.

## Supporting information


**Appendix S1.** Supporting information.

## Data Availability

Individual participant data will be shared in datasets in a de‐identified/anonymized format. Datasets from Novo Nordisk sponsored clinical research completed after 2001 for product indications approved in both the European Union and United States will be available, as well as study protocol and redacted Clinical Study Report, according to Novo Nordisk data‐sharing commitments. The data will be available permanently after research completion and approval of product and product use in both the European Union and United States, with no end date. The data may be shared with bona fide researchers submitting a research proposal requesting access to data, for use as approved by the Independent Review Board (IRB) according to the IRB Charter (see novonordisk-trials.com). An access request proposal form and the access criteria can be found at novonordisk-trials.com. The data will be made available on a specialized SAS data platform.
