# Supplementary material for: Fast‐acting insulin aspart in people with type 2 diabetes: Earlier onset and greater initial exposure and glucose‐lowering effect compared with insulin aspart
Source: Diabetes Obes Metab. 2019 Jun 10;21(9):2068–75. doi: 10.1111/dom.13767 (PMC6771872; doi:10.1111/dom.13767)
Supplement: Supplementary file 1 — Appendix S1. Supporting information. [file DOM-21-2068-s001.pdf]

Supplementary appendix

Fast-acting insulin aspart in people with type 2 diabetes: Earlier onset and greater initial exposure and glucose-lowering effect compared with insulin aspart

Authors:

Thomas R. Pieber MD[1], Eva Svehlikova MD[1], Martina Brunner MSc[2], Inge B. Halberg PhD[3], Karen Margrete Due Thomsen MSc[4], Hanne Haahr PhD[3]

Affiliations:

[1]Division of Endocrinology and Diabetology, Department of Internal Medicine, Medical University of Graz, Graz, Austria

[2]CF Clinical Research Center, Center for Medical Research, Medical University of Graz, Graz, Austria

[3]Novo Nordisk, Søborg, Denmark

[4]Novo Nordisk, Aalborg, Denmark

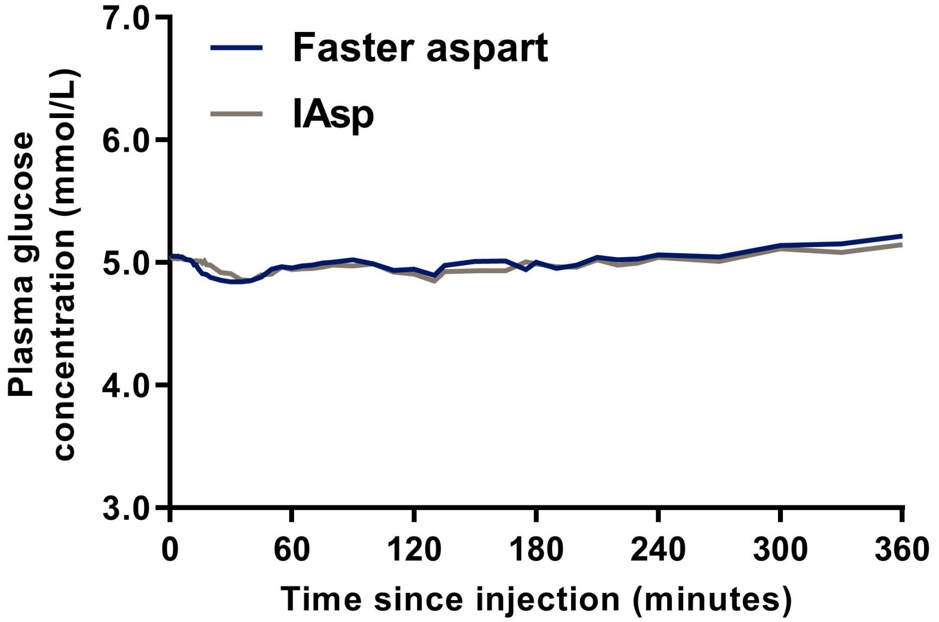

Figure S1. Mean plasma glucose concentration during a euglycaemic clamp after subcutaneous dosing of 0.3 U/kg faster aspart or IAsp in people with type 2 diabetes. Number of participants: 56 for faster aspart and 59 for IAsp.

IAsp, insulin aspart.

Table S1. Clamp quality for faster aspart and IAsp.

	Faster aspart<br>Mean±SD	IAsp<br>Mean±SD
Precision[†] (%)	3.6±0.9	3.6±0.9
Control deviation[‡] (mmol/L)	0.14±0.04	0.14±0.04

[†] The coefficient of variation of the PG measurements.
[‡] The mean difference between the PG measurements and the target blood glucose level.
Number of participants: 56 for faster aspart and 59 for IAsp.
PG, plasma glucose; SD, standard deviation.

Table S2. Pharmacokinetic blood sampling scheme.

Nominal time	IAsp	Nominal time	IAsp
00:00[a]	X	01:10	X
00:02	X	01:15	X
00:04	X	01:20	X
00:06	X	01:30	X
00:08	X	01:40	X
00:10	X	01:50	X
00:12	X	02:00	X
00:14	X	02:15	X
00:16	X	02:30	X
00:18	X	02:45	X
00:20	X	03:00	X
00:25	X	03:30	X
00:30	X	04:00	X
00:35	X	05:00	X
00:40	X	06:00	X
00:45	X	07:00	X
00:50	X	08:00	X
00:55	X	10:00	X
01:00	X	12:00	X
01:05	X		

[a] Pre-dose, i.e. within 2 minutes prior to dosing.
IAsp, insulin aspart.

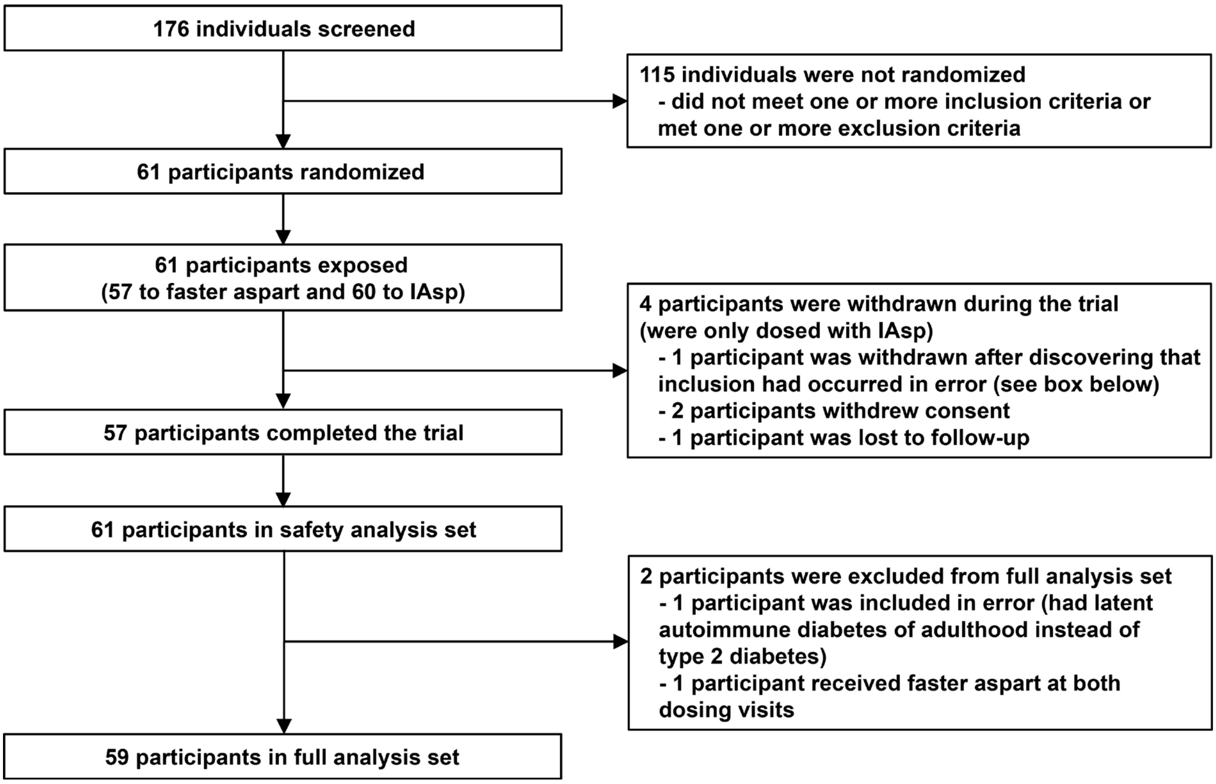

Figure S2. Participant disposition.

IAsp, insulin aspart.

Table S3. Offset of exposure and offset of glucose-lowering effect for faster aspart versus IAsp in people with type 2 diabetes.

	Faster aspart[†]	IAsp[†]	Treatment ratio[‡] [95% CI]	Treatment difference[§] [95% CI]	P-value[¶]
**Offset of exposure**					
$t_{Late\ 50\%\ Cmax}$ (min)	201.4	237.8	0.85 [0.78;0.92]	-36.4 [-55.3;-17.6]	<0.001
$AUC_{IAsp,2-t}$ (pmol·h/L)	736	841	0.88 [0.81;0.95]		0.002
**Offset of glucose-lowering effect**					
$t_{Late\ 50\%\ GIRmax}$ (min)	283.3	297.7	0.95 [0.89;1.02]	-14.4 [-34.4;5.5]	0.152
$AUC_{GIR,2-t}$ (mg/kg)	620	681	0.91 [0.82;1.01]		0.083

[†] Data are least squares means.
[‡] Faster aspart/IAsp (calculated using Fieller's method for $t_{Late\ 50\%\ Cmax}$ and $t_{Late\ 50\%\ GIRmax}$).
[§] Faster aspart - IAsp.
[¶] For treatment comparison of faster aspart versus IAsp (estimated from the linear mixed model).
Number of participants: 56 for faster aspart and 59 for IAsp.
AUC, area under the curve; CI, confidence interval; GIR, glucose infusion rate; IAsp, insulin aspart; min, minutes; $t_{Late\ 50\%\ Cmax}$, time to 50% of maximum IAsp concentration in the late part of the pharmacokinetic profile; $t_{Late\ 50\%\ GIRmax}$, time to 50% of maximum glucose infusion rate in the late part of the glucose infusion rate profile.

Table S4. Overall exposure and overall glucose-lowering effect for faster aspart versus IAsp in people with type 2 diabetes.

	Faster aspart[†]	IAsp[†]	Treatment ratio[‡] [95% CI]	P-value[§]
**Overall exposure**				
$AUC_{IAsp,0\text{-}t}$ (pmol·h/L)	1387	1403	0.99 [0.94;1.04]	0.646
C_{max} (pmol/L)	433	384	1.13 [1.02;1.24]	0.018
**Overall glucose-lowering effect**				
$AUC_{GIR,0\text{-}t}$ (mg/kg)	902	904	1.00 [0.92;1.08]	0.960
GIR_{max} (mg/kg/min)	4.0	3.9	1.03 [0.96;1.11]	0.373

[†] Data are least squares means.
[‡] Faster aspart/IAsp.
[§] For treatment comparison of faster aspart versus IAsp (estimated from the linear mixed model).
Number of participants: 56 for faster aspart and 59 for IAsp.
AUC, area under the curve; CI, confidence interval; C_{max}, maximum IAsp concentration; GIR, glucose infusion rate; GIR_{max}, maximum glucose infusion rate; IAsp, insulin aspart.
